# Kinetic Parameter Estimation and Mathematical Modelling of Lipase Catalysed Biodiesel Synthesis in a Microreactor

**DOI:** 10.3390/mi10110759

**Published:** 2019-11-08

**Authors:** Martin Gojun, Lucija Pustahija, Ana Jurinjak Tušek, Anita Šalić, Davor Valinger, Bruno Zelić

**Affiliations:** 1 University of Zagreb, Faculty of Chemical Engineering and Technology, Marulićev trg 19, HR-10000 Zagreb, Croatia; mgojun@fkit.hr (M.G.); lpustahij@fkit.hr (L.P.); bzelic@fkit.hr (B.Z.); 2 University of Zagreb, Faculty of Food Technology and Biotechnology, Pierottijeva 6, HR-10000 Zagreb, Croatia; atusek@pbf.hr (A.J.T.); dvalinger@pbf.hr (D.V.)

**Keywords:** lipase, biodiesel, microreactor, mathematical modelling

## Abstract

Development of green, clean, and sustainable processes presents new challenges in today’s science. Production of fuel is no exception. Considering the utilisation of various renewable sources, the synthesis of biodiesel, characterised as more environmentally-friendly then fossil fuel, has drawn significant attention. Even though the process based on chemical transesterification in a batch reactor still presents the most used method for its production, enzyme catalysed synthesis of biodiesel in a microreactor could be a new approach for going green. In this research, edible sunflower oil and methanol were used as substrates and lipase from *Thermomyces lanuginosus* (Lipolase L100) was used as catalyst for biodiesel synthesis. Experiments were performed in a polytetrafluoroethylene (PTFE) microreactor with three inlets and in glass microreactors with two and three inlets. For a residence time of 32 min, the fatty acids methyl esters (FAME) yield was 30% higher than the yield obtained for the glass microreactor with three inlets. In comparison, when the reaction was performed in a batch reactor (*V* = 500 mL), the same FAME yield was achieved after 1.5 h. In order to enhance the productivity of the process, we used proposed reaction kinetics, estimated kinetic parameters, and a mathematical model we developed. After validation using independent experimental data, a proposed model was used for process optimization in order to obtain the highest FAME yield for the shortest residence time.

## 1. Introduction

Applications of some traditional methods like micro-emulsification, pyrolysis, blending, or mostly transesterification, and alkaline catalysis [1,2,3,4,5] for biodiesel production are well known and established. Although these processes are common and well investigated, there are still many disadvantages that could not be solved. Some of them are problems with catalyst recovery, low quality of glycerine created as a by-product of the reaction, consumption of high quantities of energy, generation of large amounts of wastewater, etc. [6,7]. In order to solve them, there is a continuous search for new production approaches and new technologies. Some of them are based on ultrasound technology [5], supercritical solvents [8,9], or simply on novel reactor design [10,11]. On the other hand, some studies suggest that the use of enzymes (particularly lipases) for the transesterification process could be a good alternative [12]. Using this approach, energy consumption would be reduced compared to alkaline catalysis, the purity of the produced biodiesel would be higher, no soap would be formed, mild reaction conditions would be ensured, and easy catalyst recovery, if an immobilized enzyme is used, would be ensured, etc. [10]. This all makes the proposed process “greener”, which is the necessary demand in order to reduce the ecological footprint of the industry [13]. As mentioned, aside from changing the production approach, another possibility is to change the technology. According to Budžaki et al. [14], the ideal process would be a continuous process that can eliminate and/or minimize the separation and purification steps. The mentioned authors claim that this overall technology is still waiting to be developed. Perhaps a step in that direction would be the application of continuous reactors that allow the purification and separation on a single unit. Microreactors are definitely such a new technology; reactors that enhance mass and heat transfer, increase reaction rates, reduce cost and energy consumption, generate lower waste streams, etc. [12,14]. Due to their design flexibly, different variations of microreactors have been used for biodiesel synthesis [15,16,17,18], but they were mostly focused on the use of a chemical catalyst [19,20,21,22,23]. Despite the fact that enzymatic catalysis offers many advantages, it is still not competitive with the chemical process. In order to become competitive, several challenges have to be resolved. The first one is the price of the enzyme. This could be resolved by the application of suspended or immobilized enzymes, for example the use of suspended/immobilized lipase from *Thermomyces lanuginosus* (TlL), which happens to be among the least expensive commercially available lipases [24]. The cost of the process could be even lower if raw, non-purified enzymes are used. The second challenge is time. Enzymatic reactions are usually slower than chemical reactions, so the reaction rate, along with the transfer of mass and heat, need to be enhanced. As mentioned, this problem could also be resolved with the application of microreactor technology [25,26]. The third challenge, as for all new processes, is scaling-up. The process itself requires intensive work and calculations for each step, going from the laboratory to the industry, usually with a continuous decrease of productivity by increasing volume. Also, complex scaling-up procedures can be avoided by using microreactors. In order to increase throughput when working with microreactors, there is no traditional dimension/size enhancement. By using external or internal numbering up (increasing the number of microreactors), capacities of microreactors can be enhanced significantly [27,28]. Another major advantage is related to the modelling of the system. Once the process is optimised on a single chip, all the process characteristics remain the same even after capacity enlargement/numbering-up.

In summary of everything mentioned above, it can be concluded that the application of microreactor technology in combination with the lipase from TlL could be a good alternative for biodiesel production catalysed with chemical catalysts on a macro scale.

That possibility was explored in this study. A polytetrafluoroethylene (PTFE) microreactor and two different glass microreactor configurations, with two different inlet strategies, were studied. The first glass microreactor was the one with two Y-shaped inlets that allowed the introduction of a stable emulsion of methanol and oil as one phase and the enzyme as the second phase. The second glass microreactor and a PTFE microreactor consisted of three Ψ-shaped inlets, where inlets were used to separately feed in three components, methanol, oil, and enzyme dissolved in buffer, respectively. Yields obtained in all reactor configurations were compared with results obtained in a batch reactor. In order to optimise the process, kinetic parameters were estimated and a 2D mathematical model composed of convection, diffusion, and kinetic terms was developed and used. The proposed model was also used to explore the influence of oil to methanol ratio, enzyme concentration, and numbering-up (the higher residence time) on overall process efficiency. Additionally, a 2D model for three-phase flow in a microchannel using the COMSOL Multiphysics computational fluid dynamics (CFD) package (4.3b, COMSOL, Inc., Burlington, MA, USA) was developed for biodiesel production. The objective was to investigate the reactive flow and to study the velocity properties of the three phases (oil, water, and methanol) that were introduced into the microchannel with the diameter of 1 mm.

## 2. Materials and Methods

### 2.1. Materials

#### Chemicals

Edible sunflower oil (Zvijezda, Croatia) was purchased from a nearby supermarket. The lipase from *Thermomyces lanuginosus* (Lipolase 100L), FAME. mix GLC-10, sodium dodecyl sulfate (SDS), isoamyl alcohol and iso-octane were purchased from Sigma-Aldrich Handels GmbH (Vienna, Austria). Tris(hydroxymethyl)aminomethane (TRIS), methanol, HCl, were purchased from BDH Prolabo (VWR, Lutterworth, UK). Chloroform and acetonitrile were purchased from Fisher Chemicals (Loughborough, UK). 4-nitrophenyil-acetate was purchased from Acros Organics (Fischer Scientific, Merelbeke, Belgium). Potassium dihydrogen phosphate (KH_2_PO_4_) and KOH were purchased from Lach:ner (Neratovice, Czech Republic). Dipotassium hydrogen phosphate (K_2_HPO_4_) was purchased from Merck (Darmstadt, Germany).

### 2.2. Methods

#### 2.2.1. Lipase Assay

Enzyme activity was determined by a test based on hydrolysis of 1.5 mol/L 4-nitrophenyl acetate. A total of 100 μL of the sample was added to 3900 μL of TRIS-HCl buffer and homogenized. 950 μL of this mixture was added to the UV cuvette. The test is started by adding 50 μL of 1.5 mol/L 4-nitrophenyl acetate (dissolved in acetonitrile). To determine the enzyme activity, spectrophotometer (Shimadzu UV–1601, Kyoto, Japan) was used. Determination time was 60 s, while the change of absorbance was measured at 400 nm. To confirm repeatability, all measurements were performed in triplicate. On 95% confidence interval, the results showed no significant difference.

#### 2.2.2. Emulsion Preparation

The initial concentration of the SDS emulsifier was 0.1 g/L. The emulsion was prepared by mixing oil with enzyme dissolved in buffer in 8:1 ratio, and SDS was added as the selected emulsifier. The mixture was mixed on the laboratory shaker (Tehtnica, Vibromix 313EVT, Železnik Slovenia) for 15 min at 600 rpm.

#### 2.2.3. Measurement of Fatty Acid Methyl Esters (FAME) and Glycerol Concentrations

In order to determine FAME concentration method described elsewhere was used [29]. The samples were prepared for analysis by gas chromatography (Shimadzu GC-2014, Tokyo, Japan) equipped with FID and Zebron ZB-wax GC capillary column (length 30 m, I.D. 0.53 mm and film thickness 1.00 μm, Phenomenex, Torrance, CA, USA). Carrier gas in this method was helium, at rate of 1.97 mL/min. In the method’s total determination time of 15 min, measurement starts at the temperature of 180 °C for 1 min, after which at a rate of 5 °C/min, column is heating up to 230 °C. In order to identify peaks for corresponding esters of fatty acids, standard FAME mix GLC-10 was used. Retention times of fatty acids esters are as follows: 7.74 min for palmitic, 10.590 min for stearic, 10.867 min for oleic, 11.575 min for linoleic, and 12.615 min for linoleic. Glycerol determination was made with the same method and its retention time was 9.02 min. To confirm repeatability, all measurements were performed in triplicate. On 95% confidence interval, the results showed no significant difference.

#### 2.2.4. Biodiesel Synthesis in a Batch Reactor

Biodiesel synthesis in a batch reactor was performed according to procedure described by Budžaki et al. [29]. Briefly, the reaction was performed in a batch reactor (*V* = 250 mL) by adding the enzyme (45 g of Lipolase 100 L stock solution diluted with 0.01 mol/L phosphate buffer at pH 7.4 in molar ratio 1:10) in to the reaction mixture comprised of 450 g of oil and 55.95 g of methanol to form 1:3.4 molar ratio. During the experiment optimal temperature of 40 °C was maintained by a water bath with a heat regulation system (Thermomix 1420, Braun, Germany). Samples of biodiesel were taken at different time intervals and the reaction was stopped by mixing the sample with an organic solvent (Marmur solution, chloroform:isoamyl alcohol = 24:1) cooled on ice. Collected samples were then filtered (Chromafil^®^AO-20/3; 0.2 μm, Macherey, Nagel GmbH, Düren, Germany) and analysed by gas chromatography.

#### 2.2.5. Biodiesel Synthesis in Glass and PTFE Microreactors

**Figure 1:**
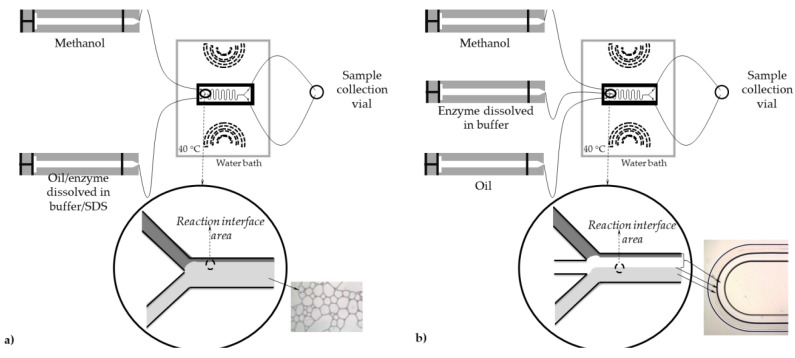
Schematic diagram of the microreactor system with (**a**) two inlets and (**b**) three inlets together with picture of formed flow pattern.

In this work, three different reactors, a PTFE coil microreactor with three inlets (+-shape; length:width = 500 mm:1 mm with an internal volume of 392.5 μL) and two glass microreactors with different inlet configurations (Y-shape and Ψ-shape inlets, respectively) of microreactors (length: width:depth = 330 mm:250 μm:50 μm with an internal volume of 4.2 μL; Micronit Microfluidics B.V., Netherlands) were examined. In the first experimental set-up (Figure 1a), emulsion which was formed from oil and enzyme dissolved in buffer (in 8:1 ratio), with the addition of emulsifier, was placed into one syringe, while the second syringe was filled with methanol. In the second and third experimental set-up, for PTFE and glass microreactor with three inlets, all substrates were placed separately into stainless steel high-pressure syringes (8 mL, Harvard Apparatus, Holliston, MA, USA), first containing oil, second enzyme dissolved in buffer (0.01 mol/L potassium phosphate buffer pH 7.4) and a third one containing methanol (Figure 1b). Syringes were places on pumps (PHD 4400 Syringe Pump Series, Harvard Apparatus, Holliston, MA, USA). Syringes were connected with silica/PTFE tubes to both microreactors. In order to obtain optimal enzyme activity experiments were performed at 40 °C. This condition was secured by submerging the microreactor in a water bath with a heat regulation system (Thermomix 1420, Braun, Germany).

In all experiments, total flows were changed in an oil:methanol:enzyme ratio 10:1.24:1 so the influence on FAME formation could be monitored. Output streams from all microreactors, that contained remained substrates (oil, methanol, enzyme) and products (FAME, glycerol), were collected in vials. In order to stop the reaction via enzyme deactivation at the exit of the microreactor, outgoing silicate tubes were emerged in an organic solvent (Marmur solution, chloroform:isoamyl alcohol = 24:1) cooled on ice.

#### 2.2.6. Kinetic Parameter Estimation

The kinetics of the reactions was investigated using the initial reaction rate method in a PTFE microreactor. The influence of each reaction compound on the initial reaction rate was monitored by keeping the concentrations of other compounds constant. When the influence of fatty acids was monitored, concentration of methanol was set to be a constant (*γ*_i, methanol_ = 40.36 mg/L) while the concentration of fatty acids was varied in the range 0–1488.53 mg/L. In the experiment where influence of methanol concertation on reaction rate was monitored, fatty acids concentration was set to be a constant (*γ*_i, fatty acids_ = 1,488.15 mg/L) while methanol concentration was changed in the rage 0–40.36 mg/L.

#### 2.2.7. Data Processing

The kinetic parameters were estimated by non-linear regression analysis from data collected from experiments in a microreactor. A software package SCIENTIST (MicroMath Scientist^®^, 3.0, MicroMath Scientific Software, Salt Lake City, UT, USA) with incorporated least square method was used to estimate the numerical values by fitting the kinetic model to the experimental data.

Mathematica 10 (Wolfram Research, Champaign, IL, USA) codes were developed and used for reactor model simulation and verification.

The CFD of the oil, methanol, and buffer phase was carried out using the finite element software COMSOL Multiphysics v. 4.3b.

### 2.3. Mathematical Modelling

#### 2.3.1. Modelling of Biodiesel Transesterification

For the description and prediction of the biodiesel transesterification process in a microreactor 2D model was developed [30,31] considering convection in the flow (*x*) direction, diffusion in two directions (*x* and *y*) and kinetics of enzyme catalysed reaction (Michaelis–Menten kinetics).

Dimensionless partial differential equations for steady-state conditions in the single pass microreactor system with the associated boundary conditions are as follows (Equations (1)–(6)):

- Enzyme in aqueous phase (phase 1):
  (1) $$v_{1}\cdot \frac{\partial \gamma_{E,1}}{\partial \xi }=\frac{D_{E/\mathrm{aq}}}{W}\cdot \left(\frac{\partial^{2}\gamma_{E,1}}{\partial \xi^{2}}+\frac{\partial^{2}\gamma_{E,1}}{\partial \psi^{2}}\right)$$
  $\gamma_{E,1}\left(0,\psi \right)=\gamma_{E,1,i},\;\;-1\leq \psi \leq 0$
  $\frac{\partial \gamma_{E,1}\left(\frac{L}{W},\psi \right)}{\partial \xi }=0,\;\;-1\leq \psi \leq 0$
  $\gamma_{E,1}\left(\xi ,0\right)=K_{P,E}\cdot \gamma_{E,2}\left(\xi ,0\right),\;\;0<\xi <\frac{L}{W}$
  $\frac{\partial \gamma_{E,1}\left(\xi ,1\right)}{\partial \psi }=0\;,\;\;0<\xi <\frac{L}{W}$
- Enzyme in oil phase (phase 2):
  (2) $$v_{2}\cdot \frac{\partial \gamma_{E,2}}{\partial \xi }=\frac{D_{E/\mathrm{oil}}}{W}\cdot \left(\frac{\partial^{2}\gamma_{E,2}}{\partial \xi^{2}}+\frac{\partial^{2}\gamma_{E,2}}{\partial \psi^{2}}\right)$$
  $\gamma_{E,2}\left(0,\psi \right)=0,\;\;0\leq \psi \leq 1$
  $\frac{\partial \gamma_{E,2}\left(\frac{L}{W},\psi \right)}{\partial \xi }=0,\;\;0\leq \psi \leq 1$
  $\frac{\gamma_{E,2}\left(\xi ,0\right)}{\partial \psi }=\frac{D_{E/\mathrm{aq}}}{D_{E/\mathrm{oil}}}\cdot \frac{\gamma_{E,1}\left(\xi ,0\right)}{\partial \psi },\;\;0<\xi <\frac{L}{W}$
  $\frac{\partial \gamma_{E,2}\left(\xi ,1\right)}{\partial \psi }=0,\;\;0<\xi <\frac{L}{W}$
- Methanol in aqueous phase (phase 1):
  (3) $$v_{1}\cdot \frac{\partial \gamma_{M,1}}{\partial \xi }=\frac{D_{M/\mathrm{aq}}}{W}\cdot \left(\frac{\partial^{2}\gamma_{M,1}}{\partial \xi^{2}}+\frac{\partial^{2}\gamma_{M,1}}{\partial \psi^{2}}\right)-W\cdot V_{max}\cdot \frac{\gamma_{E,1}\cdot \gamma_{M,1}\cdot \gamma_{\mathrm{FA},1}}{\left(K_{m}^{M}+\gamma_{M,1}\right)\cdot \left(K_{m}^{\mathrm{FA}}+\gamma_{\mathrm{FA},1}\right)}$$
  $\gamma_{M,1}\left(0,\psi \right){=}_{M,1,i},\;\;-1\leq \psi \leq 0$
  $\frac{\partial \gamma_{M,1}\left(\frac{L}{W},\psi \right)}{\partial \xi }=0,\;\;-1\leq \psi \leq 0$
  $\gamma_{M,1}\left(\xi ,0\right)=K_{P,M}\cdot_{M,2}\left(\xi ,0\right),\;\;0<\xi <\frac{L}{W}$
  $\frac{\partial \gamma_{M,1}\left(\xi ,1\right)}{\partial \psi }=0,\;\;0<\xi <\frac{L}{W}$
- Methanol in oil phase (phase 2):
  (4) $$v_{2}\cdot \frac{\partial \gamma_{M,2}}{\partial \xi }=\frac{D_{M/\mathrm{oil}}}{W}\cdot \left(\frac{\partial^{2}\gamma_{M,2}}{\partial \xi^{2}}+\frac{\partial^{2}\gamma_{M,2}}{\partial \psi^{2}}\right)-W\cdot V_{max}\cdot \frac{\gamma_{E,2}\cdot \gamma_{M,2}\cdot \gamma_{\mathrm{FA},2}}{\left(K_{m}^{M}+\gamma_{M,2}\right)\cdot \left(K_{m}^{\mathrm{FA}}+\gamma_{\mathrm{FA},2}\right)}$$
  $\gamma_{M,2}\left(0,\psi \right)=0,\;\;0\leq \psi \leq 1$
  $\frac{\partial \gamma_{M,2}\left(\frac{L}{W},\psi \right)}{\partial \xi }=0,\;\;0\leq \psi \leq 1$
  $\frac{\gamma_{M,2}\left(\xi ,0\right)}{\partial \psi }=\frac{D_{M/\mathrm{aq}}}{D_{M/\mathrm{oil}}}\cdot \frac{\gamma_{M,1}\left(\xi ,0\right)}{\partial \psi },\;\;0<\xi <\frac{L}{W}$
  $\frac{\partial \gamma_{M,2}\left(\xi ,1\right)}{\partial \psi }=0,\;\;0<\xi <\frac{L}{W}$
- Fatty acids in aqueous phase (phase 1):
  (5) $$v_{1}\cdot \frac{\partial \gamma_{\mathrm{FA},1}}{\partial \xi }=\frac{D_{\mathrm{FA}/\mathrm{aq}}}{W}\cdot \left(\frac{\partial^{2}\gamma_{\mathrm{FA},1}}{\partial \xi^{2}}+\frac{\partial^{2}\gamma_{\mathrm{FA},1}}{\partial \psi^{2}}\right)-W\cdot V_{max}\cdot \frac{\gamma_{E,1}\cdot \gamma_{M,1}\cdot \gamma_{\mathrm{FA},1}}{\left(K_{m}^{M}+\gamma_{M,1}\right)\cdot \left(K_{m}^{\mathrm{FA}}+\gamma_{\mathrm{FA},1}\right)}$$
  $\gamma_{\mathrm{FA},1}\left(0,\psi \right)=0,\;\;-1\leq \psi \leq 0$
  $\frac{\partial \gamma_{\mathrm{FA},1}\left(\frac{L}{W},\psi \right)}{\partial \xi }=0,\;\;-1\leq \psi \leq 0$
  $\frac{\gamma_{\mathrm{FA},1}\left(\xi ,0\right)}{\partial \psi }=\frac{D_{\mathrm{FA}/\mathrm{oil}}}{D_{\mathrm{FA}/\mathrm{aq}}}\cdot \frac{\gamma_{\mathrm{FA},2}\left(\xi ,0\right)}{\partial \psi },\;\;0<\xi <\frac{L}{W}$
  $\frac{\partial \gamma_{\mathrm{FA},1}\left(\xi ,1\right)}{\partial \psi }=0,\;\;0<\xi <\frac{L}{W}$
- Fatty acids in oil phase (phase 2):
  (6) $$v_{2}\cdot \frac{\partial \gamma_{\mathrm{FA},2}}{\partial \xi }=\frac{D_{\mathrm{FA}/\mathrm{oil}}}{W}\cdot \left(\frac{\partial^{2}\gamma_{\mathrm{FA},2}}{\partial \xi^{2}}+\frac{\partial^{2}\gamma_{\mathrm{FA},2}}{\partial \psi^{2}}\right)-W\cdot V_{max}\cdot \frac{\gamma_{E,2}\cdot \gamma_{M,2}\cdot \gamma_{\mathrm{FA},2}}{\left(K_{m}^{M}+\gamma_{M,2}\right)\cdot \left(K_{m}^{\mathrm{FA}}+\gamma_{\mathrm{FA},2}\right)}$$
  $\gamma_{\mathrm{FA},2}\left(0,\psi \right){=}_{\mathrm{FA},2,i},\;\;0\leq \psi \leq 1$
  $\frac{\partial \gamma_{\mathrm{FA},2}\left(\frac{L}{W},\psi \right)}{\partial \xi }=0,\;\;0\leq \psi \leq 1$
  $\frac{\gamma_{\mathrm{FA},2}\left(\xi ,0\right)}{\partial \psi }=K_{P,\mathrm{FA}}\cdot_{\mathrm{FA},1}\left(\xi ,0\right),\;\;0<\xi <\frac{L}{W}$
  $\frac{\partial \gamma_{\mathrm{FA},2}\left(\xi ,1\right)}{\partial \psi }=0,\;\;0<\xi <\frac{L}{W}$

A systems of partial differential equations were solved by using the 2D finite differences method. Partial derivatives were discretised on the static equidistant grid.

The diffusion coefficient (*D*_S/B_) of methanol at 40 °C, was estimated according to Scheibel empirical correlation (Equation (7)) proposed by Li and Carr [32]:

(7) $$D_{S/B}=\frac{8.2\cdot 10^{-8}\cdot T}{\eta_{B}\cdot V_{S}^{1/3}}{\left[1+\left(\frac{3\cdot V_{B}}{V_{S}}\right)\right]}^{2/3}$$

The diffusion coefficient (*D*_S/B_) of enzyme at *T* = 40 °C, was estimated according to Young empirical correlation [33] (Equation (8)):

(8) $$D_{S/B}=8.34\cdot 10^{-8}\left(\frac{T}{\eta_{B}\cdot M_{S}^{1/3}}\right)$$

Properties of the solutes and solvents that were used for calculations are presented in Table 1.

Additionally, the diffusion times of components diffusing from one phase to another were calculated according to Equation (9):

(9) $$\tau_{D}=\frac{W^{2}}{D_{S/B}}$$

where *W* denotes width that components have to cross from one phase to another.

#### 2.3.2. CFD Modelling

COMSOL Multiphysics 4.3b software was used for the solving of partial differential equations in order to obtain 2D velocity model of multiphase laminar flow in microchannel since COMSOL Multiphysics uses the finite element method together with adaptive meshing and error control using a variety of numerical solvers. The meshing is regarded to geometry division which is often triangular shaped (software default meshing) which can be modified to any shape or size by user. For this study the model was developed for room temperature conditions and the Navier–Stokes equations for incompressible fluids along with the continuity equation were used to model multiphase flow. The boundary conditions, that are commonly used, were also assumed for this model: no-slip at the walls, fully developed laminar flow, defined velocities for the inflow of each phase, and zero relative pressure for the outflow. The Navier–Stokes equations for the conservation of momentum used (Equation (10)):

(10) $$\rho \frac{\partial u}{\partial t}+\rho u\cdot \nabla u=-\nabla p+\nabla \cdot (u(\nabla u+{\left(\nabla u\right)}^{T})+F_{v}$$

and the continuity equation for conservation of mass (Equation (11)):

(11) $$\frac{\partial \rho }{\partial t}+\nabla \cdot (\rho u)=0$$

For the meshing one of the default options that the Comsol Multiphysics 4.3b provides was chosen in term of physics controlled finer triangular mesh which consisted of 48,138 domain elements and 4850 boundary elements since preliminary results with normal grid which consisted of 30804 domain elements and 3877 boundary elements gave less accurate results. Additionally, extra fine and extremely fine mesh were tested and the results showed no difference from the finer mesh which was selected.

## 3. Results and Discussion

Biodiesel was produced from edible sunflower oil, using the enzyme Lipolase 100L (*V.A.* > 100,000 U/mL), in a batch reactor (*V* = 250 mL), a PTFE (length:width = 500 mm: 1 mm with an internal volume of 392.5 μL), and a glass microreactor (length:width:depth = 330 mm:250 μm:50 μm with an internal volume of 4.2 μL) with two different microreactor inlet configurations. Based on previous experiments, every configuration has some pros and cons, so the first step was to examine which configuration yields better FAME and, consequently, which one is economically more viable (lower usage of substrates, shorter preparation of experimental set-up, and implementation).

### 3.1. Biodiesel Production

**Figure 2:**
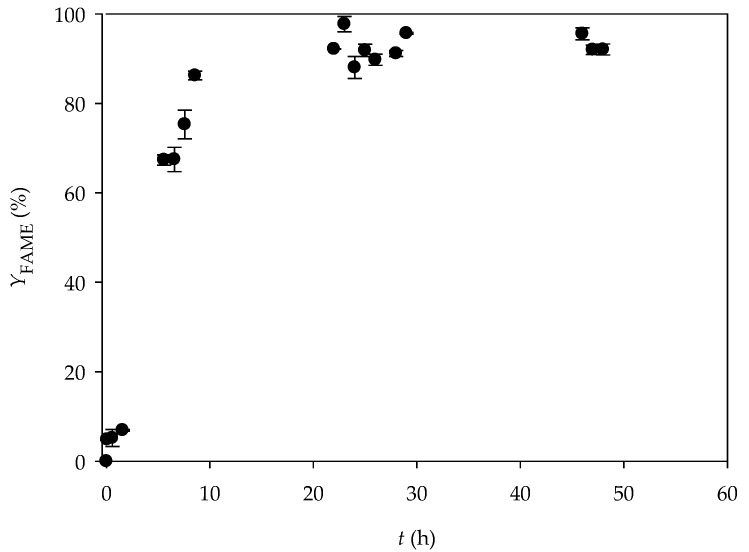
Lipase catalysed biodiesel synthesis in a batch reactor.

First, the biodiesel was synthetized in a batch reactor by repeating the experimental process described in the paper by Budžaki et al. [29]. As can be seen from Figure 2, the highest fatty acids methyl esters (FAME) yield of 97.70% ± 1.49 was achieved after 24 h in the one-step transesterification reaction of edible sunflower oil performed at 40 °C. At the end of the process, the results corresponded to those obtained by Budžaki et al. [29].

The next step was production on a micro scale. As mentioned in the materials and methods section, based on two microreactor inlet configurations, it was possible to introduce the reaction components in two different ways. The first possibility was to form a stable emulsion of two components (methanol and oil, buffer and oil, or methanol and buffer) and introduce them as one phase, while the third reaction component would be introduced as a separate phase. The second possibility would be to introduce each component separately.

#### 3.1.1. Two Inlets Strategy

In one of our previous studies [18], different approaches to reactant supply were investigated when a microsystem (PTFE/Teflon tubular microreactor, length:width = 500 mm:1 mm, internal volume 392.5 μL) with two inlets was used for biodiesel production. In order to introduce three components into a microreactor with two inlets, two components had to be mixed together. Three different strategies of feeding components into the microchannel were investigated. The first one was based on an enzyme and methanol mixture which was introduced as one phase, and oil was the second phase. In the second strategy, oil and methanol were mixed together using an emulsifier (SDS) and introduced into a microchannel as one phase, and the enzyme dissolved in buffer was the second phase. In the third strategy, oil and the enzyme also formed a mixture using an emulsifier (Triton X-100, Sigma-Aldrich Handels GmbH, Darmstadt, Germany) and the methanol was introduced separately. In the mentioned research, similar FAME yields were observed for all performed experiments.

Although the FAME yield was similar in all three experiments, two of the proposed systems had a disadvantage. When working with a system where methanol and the enzyme formed a mixture, it was noticed that after a longer period of activity, the methanol deactivates the enzyme (*k_d_* = 0.0037 ± 0.00002 min^−1^ [18]). Although this was not an obstacle for the short laboratory experiments, in case of the long continuous processes, this would cause a significant problem. As mentioned in that research, to resolve this problem, it was necessary to separate the methanol and the enzyme from the inlet feeding container. Since the oil is immiscible with both methanol and the enzyme/buffer, it was necessary to introduce an additional component, the emulsifier. After the formation of stable emulsions, buffer/oil/SDS, and methanol/oil/Triton X-100 experiments were performed. A system composed of methanol/oil/Triton X-100 was rejected because the emulsion was not stable enough for a longer period of time, again leading to the same problem in the case of continuous production. In the end, the system where the enzyme/oil/SDS emulsion was fed into the reactor as one of the inlet streams and methanol as the second one, was proposed as the best one.

**Figure 3:**
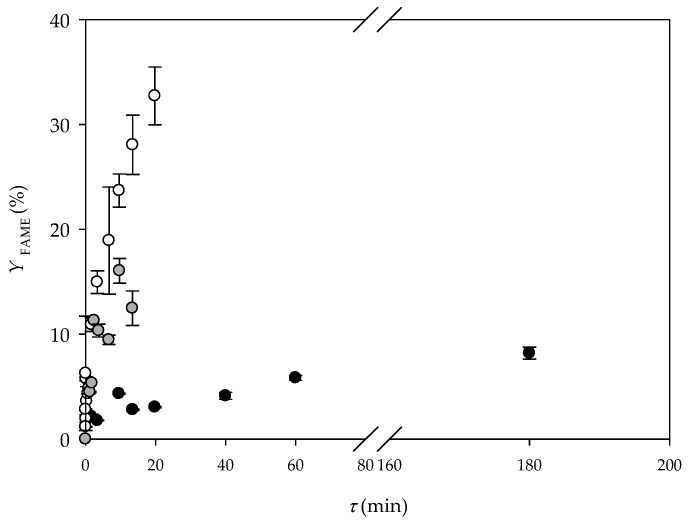
Lipase catalysed biodiesel synthesis in a glass microreactor with (**a**) two inlets (●), and (**b**) three inlets (○) and a polytetrafluoroethylene (PTFE) microreactor with tree inlets (●).

In the present study, the same strategy was applied (Figure 1a). Before the sample collection started, the flow profile was monitored under the microscope (Motic B1-220A, binocular, Weltzar, Germany). It was noticed that a mixture of oil and buffer (due to larger viscosity and density, as well as faster flow rate) occupies more than half of the microchannel volume. The flow was mostly stable and parallel during the measuring, but the frequency of the instabilities increased by increasing the residence time. Due to viscosity, the flow went from parallel to churn flow (Figure 1a). The results of the lipase catalysed biodiesel synthesis in a microreactor are presented in Figure 3. Up to 15% of the FAME yield was reached for the residence time of 4 min. The comparison with the results from the batch reactor confirmed that the reaction was 15 times faster in a microreactor, since the same FAME yield in a batch reactor was recorded after 1 h.

#### 3.2.2. Three Inlets Strategy—Glass Microreactor

As can be noticed, an additional component (SDS) was introduced into the system in the previous experiment. Even though this component does not have a negative effect on the production process (no enzyme inhibition), it presents a problem for the product purification step, since it has to be removed from the final product. An additional problem was the enzyme that was a part of the oil phase, and at the end, a part of the biodiesel. In this way, it was not possible to reuse the enzyme (i.e., via recirculation) and it also had to be removed from the final product. In order to avoid these problems, a microreactor with three inlets was proposed. All microreactor characteristics (volume, length, depth, width, and outlet) were the same as for the microreactor with two inlets, for the purpose of comparing the impact of the feeding strategy. Before the experiment was performed, it was again crucial to define the inlet strategy, which means defining the position of each component inlet.

**Figure 4:**
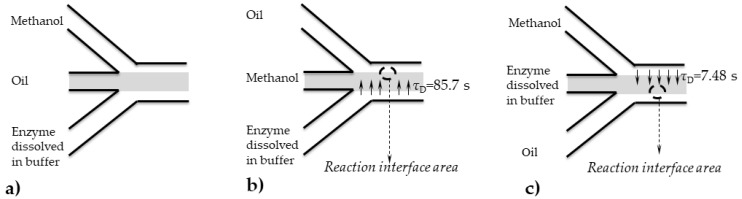
Inlet scheme with three different component inlet strategies, when (**a**) oil, (**b**) methanol, and (**c**) enzyme dissolved in buffer are fed in the middle channel.

When working with a microreactor with three inlets, there are three possibilities to introduce all three components of a reaction mixture: a) oil, b) methanol, and c) enzyme dissolved in buffer are fed in the middle channel and the other two components are fed from the edge inlets (Figure 4). In order to maximise the mass transfer, it was necessary to determine the right inlet position for all three components.

The first option (Figure 4a), when oil is fed in the middle, was rejected, since oil is immiscible with both methanol and buffer. This would create an oil barrier that would not allow methanol to diffuse to the enzyme-active site on the opposite sight of the microchannel, so the reaction would be blocked. Based on this, it was assumed that a significant mass transfer will take place only through the interface area between the methanol and the buffer phase if they are placed next to each other. Once this mixture is formed, a reaction would take place on the second interface area, formed between the mixture and the oil. In other to determine which option is better, B or C (Figure 4), diffusion coefficients were calculated using the empirical correlation (Equations (7) and (8)) and data collected from literature.

**Figure 5:**
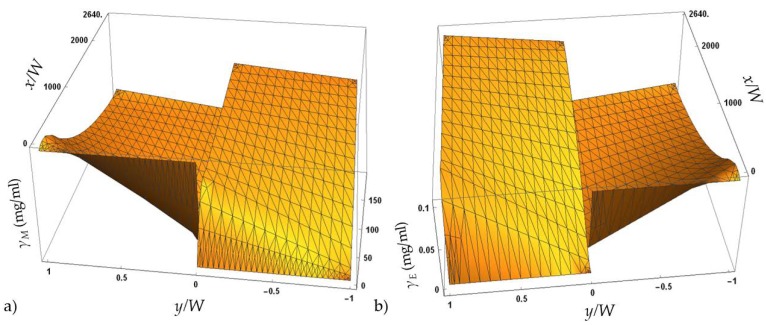
Diffusivity profile for (**a**) methanol and (**b**) enzyme in a microreactor channel for overall flow rate of *Φ* = 0.03817 µL/min.

As shown in Table 2, the diffusion coefficient for methanol was calculated to be higher than the one for the enzyme, meaning it will diffuse faster than the enzyme to another phase. Due to the size of both components, the obtained result was as expected. Additionally, combining the calculated values and partial differential equations (Equations (1)–(6)), without the kinetic part of the equation, a numerical simulation of the concentration profiles in a microreactor channel for methanol and lipase was obtained. Results confirming faster methanol diffusion are presented in Figure 5. As can be seen, the methanol diffuses almost completely to aqueous phase, even at the very beginning of the microchannel, which is important, since all the components have to be available at the interface area between the oil and the aqueous phase for the start of the reaction.

Based on the obtained results, it was determined that the proposed option c), where the enzyme dissolved in buffer was placed in the middle, and oil and methanol on edge, was the favourable option.

Before biodiesel synthesis was performed in a microreactor, and in order to calculate diffusion time, the flow profile and flow stability along the length of the microchannel were investigated. The flow profile was monitored for different flow velocities using a microscope (Motic B1-220A, binocular, Weltzar, Germany). By feeding the components in the oil:methanol:enzyme ratio of 10:1.24:1 (based on successfully performed experiments in a macroreactor published elsewhere [29]), it was noticed that the oil phase (due to different physical properties) occupies half of the microchannel volume (Figure 1b).

In order to determine the space of the microchannel occupied by each phase for different flow rates exactly, a series of pictures were taken and analysed for the position at the beginning of the microchannel. As mentioned, it was noticed that the oil phase occupies half of the microchannel width (125 μm), while the mixture of methanol and buffer occupies the other half of the microchannel (125 μm). Due to fast methanol diffusion (calculated by using Equation (9) to be 7.5 s (Table 2)), according to calculated diffusion times, for all the residence times higher than 7.5 s, a two-phase system becomes a monophase system, with a width of 125 µm. As for the flow profile, a parallel, mostly stable fluid flow was developed from the entrance to the exit of the microchannel. Due to high oil viscosity, slight instabilities were noticed in different periods, but the fluid flow quickly become stable again.

Based on all results, it can be concluded that a favourable residence time for the reaction is over 10 s. In that time, both substrates are positioned closely (on the interface area, *A* = 1.66 nm^2^, in the middle of the channel) and are available to the enzyme-active site so the reaction can be performed.

The results of the lipase catalysed biodiesel synthesis in a microreactor with three inlets are presented in Figure 3. A FAME yield of up to 32% was reached for the residence time of 20 min. In comparison, when the reaction was performed in a batch reactor (*V* = 250 mL), the same FAME yield was achieved after 1.5 h. Comparing the obtained results with the results from when a two-inlet microreactor was used, the same trend was noticed for all residence times, indicating that there is no difference in microreactor performance despite the inlet feeding strategies. Considering all the advantages of separate feeding for each component: no addition of new components, stable flow from the entrance to the exit of the microchannel, which makes the separation of biodiesel from the other component and consequently easier purification and potential enzyme recirculation possible, a microreactor with three inlets is the better choice.

#### 3.2.3. Three Inlets Strategy—PTFE Microreactor

Even though both reactions performed in glass microreactors showed promising results in comparison to the batch reactor, one major problem was noticed during both experiments. Due to the small dimensions of the microchannels, clogging occurred, making the further work impossible. According to Poe et al. [37], when working with small diameter solids (for example enzyme dispersion) in a microreactor, or with highly viscous solvents (like oil), clogging can occur. In order to resolve this problem, a PTFE tubular microreactor with a larger diameter (1 mm) was tested for biodiesel production. The same microreactor type was successfully used in one of previous experiments [18] and no clogging formation was noticed.

Based on the results obtained for the glass microreactor in this research, the three inlets strategy, where the enzyme dissolved in buffer was placed as the middle inlet stream, was implemented for the PTFE microreactor. The obtained results are presented in Figure 3. The FAME yield of 8.9% was noticed for the residence time of 3 h, which was significantly lower in comparison to both glass microreactors. The reason for this is the different hydrodynamic and mass transfer rate that increases with the reduction of the channel size. On the other hand, if the reaction is performed in a PTFE tubular microreactor, but with a high excess of methanol, a FAME yield of 98% can be obtained in 2 h [18]. This indicated that the proposed process needs additional optimization.

In order to do so, a 2D mathematical model was developed considering the convection in the flow (*x*) direction, the diffusion in two directions (*x* and *y*), and the kinetics of the enzyme catalysed reaction (Michaelis–Menten kinetics) (Equations (1)–(6)).

In order to simplify the mathematical model, some assumptions were made:

1. The reaction occurs in the interphase area between phases.
2. Even though triacylglycerols, diacylglycerols, monoacylglycerols, and free fatty acids are present in the mixture, they could be treated as a single constituent [38].
3. Methanol is the main inhibitor of the enzyme, but due to the fact that the reaction is performed in a continuously operated tubular microreactor where methanol is constantly removed from the mixture, the inhibition effect was neglected [39].
4. The limiting step of the reaction was considered to be hydrolysis, but according to literature, if the percentage of water in the process is between 2–20%, the reaction is shifted towards transesterification. In the present research, the water content was calculated to be below 8% (*w*/*w*), so the hydrolysis reaction was also neglected.
5. It was assumed that the flow is laminar and parallel from the beginning of the microchannel.

The kinetic parameters were estimated from independent experiments performed in a microreactor, the mathematical model was validated and used to estimate the best process conditions for the experiment performed in a PTFE tubular microreactor.

### 3.2. Kinetic Parameter Estimation

**Figure 6:**
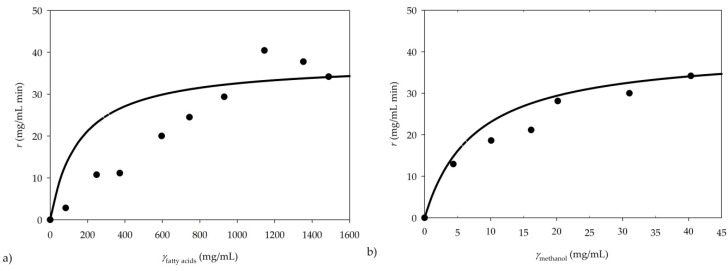
Kinetics of transesterification: dependence of the initial reaction rate on concentration of (**a**) fatty acids and (**b**) methanol.

The influence of fatty acids and the methanol concentration on the initial reaction rate was investigated to estimate the kinetic parameters of the Michaelis–Menten kinetic model. The experiments were performed for the residence time of 0.6 min in a continuously operated microreactor. The results are presented in Figure 6.

The kinetic parameters were estimated by non-linear regression, using the experimental results and double substrate Michaelis–Menten kinetics, and determined to be *V*_max_ = 44.71 ± 1.89 mg/mL min, *K*_m fatty acids_ = 155.02 ± 121.86 mg/mL and *K*_m methanol_ = 7.56 ± 2.77 mg/mL. In order to analyse the importance of individual kinetic parameters included in the model, the Fourier amplitude sensitivity test (FAST) was applied. The effects of the kinetic parameters on the reaction rate, *r*, were analysed (data not shown). It was determined that *V*_max_ has the highest effect on the reaction rate in comparison to other parameters.

The obtained kinetic parameters were compared with data from the literature. Unfortunately, there is limited data on the Michaelis–Menten enzyme kinetics of the transesterification in a batch reactor using lipase from *Thermomyces lanuginosus* for biodiesel production. On the other hand, the kinetics were studied for many other lipases from different sources, but again with a different approach (mostly using Bi-Bi Ping-Pong kinetics) so a comparison was impossible. A detailed kinetic model of biodiesel production by free lipase *Callera Trans L* was described by Firdaus et al. [40], but with a different approach. On the other hand, Cheirsilp et al. [41] investigated the kinetics of transesterification of palm oil and ethanol for fatty acid ethyl ester production using Lipase PS (*Pseudomonas* sp.), Sun et al., [42] studied the kinetics of the transesterification of palm oil and dimethyl carbonate for biodiesel production, and Vey et al. [43] analysed the kinetics of LipozymeR for the transesterification of Jatropha curcas oil.

Due to the absence of literature data, it was assumed that due to the large surface to volume ratios, interfacial interactions play a significant role in the microreactor and define the predominantly laminar flow and stronger diffusion control compared to large scale reactors. As a consequence, the kinetic constants obtained in microreactor experiments could be different from those estimated using data collected from microreactor experiments [44].

### 3.3. Velocity Model of Multiphase Laminar Flow in a Microreactor

**Figure 7:**
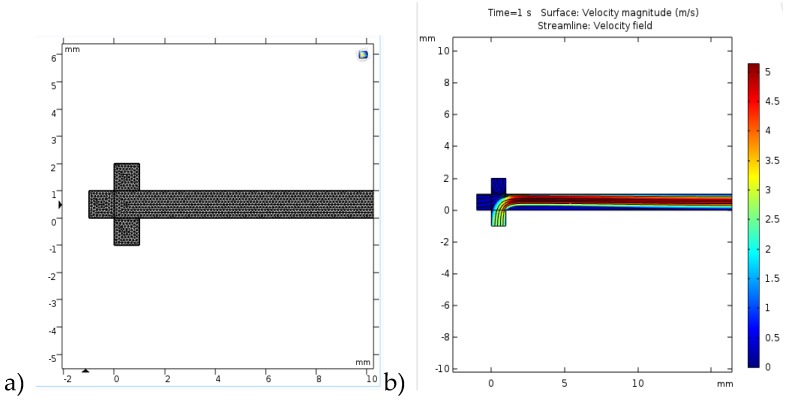
Computational fluid dynamics (CFD) simulation of velocity profile in microsystem (**a**) grid used for the simulation, and (**b**) velocity profile.

Since it was not possible to observe the flow profile under the microscope for the PTFE tube (not transparent), like it was done for the glass microreactor (Figure 1b prior to model validation, it was necessary to prove the assumption mentioned in Section 3.2.3., meaning that the flow is parallel and stable from the beginning of the microchannel. As mentioned before, for the meshing, one of the default options provided by the Comsol Multiphysics was chosen (Figure 7a), in terms of physics, a controlled finer triangular mesh, which consisted of 48,138 domain elements and 4850 boundary elements after preliminary results with normal a grid, which consisted of 30,804 domain elements and 3877 boundary elements, provided less accurate results. Additionally, an extra fine and an extremely fine mesh were tested, and the results showed no difference from the finer mesh which was selected. The results, in term of the velocity profile in the microchannel, are presented in Figure 7b.

Since the initial flow rates of the three phases that were introduced into the microchannel differ, the velocity profiles provided details on how they interconnect. The lower (bottom) inflow represents the oil phase with the initial flow rate of 2.6 m/s, while the left inflow is the water phase with the initial flow rate of 0.26 m/s, and the upper (top) inflow is the methanol phase with the initial flow rate of 0.33 m/s. Even though the velocity streamlines are directed to the top of the microchannel at the start of the microchannel due to the fact that the oil phase had almost 10-fold higher initial value, after a certain length of microchannel, an almost normal flow throughout the microchannel is observed in terms of the even distribution of velocities, slightly leaning towards the top of the microchannel, which is to be expected. This sort of a simulation can sometimes be of great importance, not only to observe the velocities, but also to see whether there are dead spaces in the microchannel, like in this case with the small portion of the lower microchannel after the oil inflow.

### 3.4. Model Validation

In order to validate the proposed mathematical model (Equations (1)–(6)), two independent experiments preformed in a microreactor were used. Two different inlet concentrations of methanol were used, where a concentration of 40.36 mg/L was introduced to the microreactor in experiment 1, and the inlet methanol concentration was 10-fold higher in experiment 2 [18].

**Figure 8:**
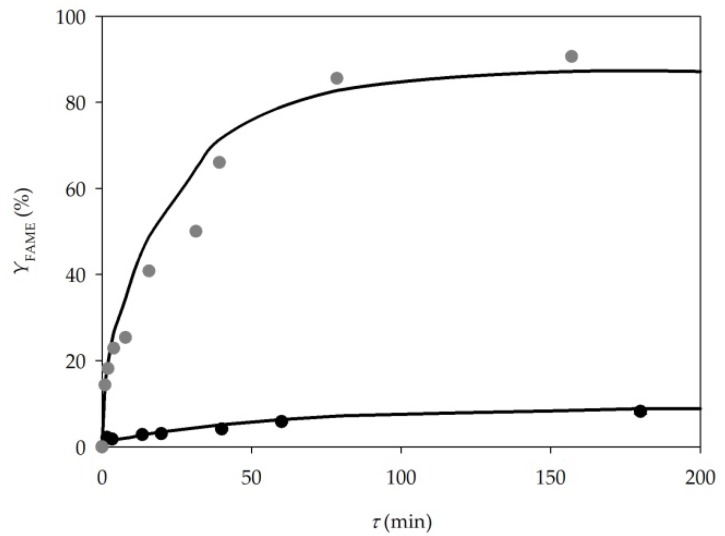
Validation of proposed mathematical model for lipase catalysed transesterification in a PTFE microreactor for different inlet methanol concentrations; 403.6 mg/L (●), 40.36 mg/L (●), model (―).

The experimentally obtained data was compared with the model simulation results and it can be noticed that the model describes the trend of the experimental data very well (Figure 8).

Based on the experimental results and the model predictions, the obtained model could be used for further process optimisation.

### 3.5. Process Optimization

The proposed mathematical model was used for the optimisation of the transesterification process catalysed by lipase, in order to collect data for further research. Model-based process optimisation is a fast and effective tool for the prediction of influence of different process parameters on process efficiency. The same approach was previously described by Santana et al. [19], where a mathematical model was used for the simulation of sunflower esterification with ethanol in the presence of sodium hydroxide.

In theory, there are several process parameters that could be altered in order to enhance the process productivity. The first one is certainly the effect of residence time. Therefore, the effect of residence time on process efficiency was analysed first. As shown in Table 3, no significant change in transesterification efficiency was noticed by prolonging residence time.

Another parameter that has an effect on process productivity is enzyme concentration. Model simulation results showed the positive influence of the enzyme concentration increase on the FAME content. An increase from 8.849% to 10.282% was achieved when the enzyme concentration in the inlet stream was increased from 0.1 mg/mL to 0.3 mg/mL. On the other hand, considering the price of the enzyme, this increase in yield is not sufficient to justify the application of larger amounts of the enzyme. 

As presented in Figure 8, by comparing the yields obtained for the molar ratio of 1:34 [18] and for the ratio of 1:3.4, it is obvious that by enhancing the oil to methanol ratio, a higher FAME yield in shorter residence time can be achieved. As can be seen from the data shown in Table 3, the increase of the molar ratio of oil to methanol has the biggest impact on the FAME yield, in comparison to other process parameters. Thus, as a reference for future work, additional research on the effect of the oil to methanol ratio is recommended.

## 5. Conclusions

In this research, edible sunflower oil and methanol were used as substrates and lipase from *Thermomyces lanuginosus* (Lipolase L100) was used as a catalyst for biodiesel synthesis. Experiments were performed in microreactors equipped with two or three inlets, and correspondingly, two different feeding strategies were used. For the residence time of 32 min, fatty acids methyl esters (FAME) yield was higher than 30% for the experiment performed in a microreactor equipped with three inlets. For comparison, when the reaction was performed in a batch reactor (*V* = 250 mL), the same FAME yield was achieved after 1.5 h. On the other hand, this microreactor type was discarded due to clogging and a PTFE tubular microreactor was proposed as a better solution for biodiesel production. The proposed mathematical model predicted the trend of enzyme catalysed transesterification in a microreactor very well and it was used for further process optimisation. The mathematical model simulation results indicate that the residence time is the most significant process parameter.

## Figures and Tables

**Table 1:** Properties of methanol, water (buffer) and lipase.

Solute (S)	Solvent (B)	*T* (K)	*V*_S_ (mL/mol)	*V*_B_ (mL/mol)	*M*_S_ (g/mol)	*η*_B_ (mPa·s)
Lipase	Water (buffer)	313.15	-	18.069 [34]	33,400 [35]	0.654
Methanol	Water (buffer)	313.15	38.5 [36]	18.069 [34]	32.04	0.654
Lipase	Methanol	313.15	-	38.5 [36]	33,400 [36]	0.445
Methanol	Methanol	313.15	38.5 [36]	38.5 [36]	32.04	0.445

**Table 2:** Estimated diffusivities and average diffusion time for methanol and lipase at 40 °C.

Solute (S)	Solvent (B)	*D*_S/B_·10^−9^ (m^2^/s)	*τ*_D_ (s)
Lipase	Water (buffer)	12.40	-
Methanol	Water (buffer)	2.09	7.48
Lipase	Methanol	18.22	85.7
Methanol	Methanol	1.71	-

**Table 3:** Optimisation of the process by model simulation (values in brackets are results obtained from laboratory experiments).

Effect of Residence Time		Effect of Enzyme Concentration		Effect of Oil to Methanol Ratio	
*τ* (min)	*Y*_FAME_ (%)	*γ*_E_ (mg/mL)	*Y*_FAME_ (%) (*τ* = 180 min)	Oil:Methanol Molar Ratio	*Y*_FAME_ (%) (*τ* = 180 min)
180	8.849 (8.18)	0.1	8.849 (8.18)	1:3.4	8.849 (8.18)
270	9.729	0.2	9.505	1:17	40.938
360	10.245	0.3	10.282	1:34	87.179 (89.56)
450	10.583	-			

## Nomenclature

*A*	Interface area (mm^2^)
*c*	Concentration (mmol/L)
*D*_S/B_	Diffusion coefficient (m^2^/s)
*F*_v_	Volume force vector (N/m^3^)
*K*_m_	Michaelis–Menten constant (mmol/L)
*L*	Microchannel length (m)
*M*_B_	Molecular weight of solvent (g/mol)
*p*	Pressure (Pa)
*T*	Temperature (°C, K)
*v*	Linear velocity (m/s)
*u*	Velocity vector (m/s)
*V*	Reactor volume (μL)
*V.A.*	Volumetric activity (U/mL)
*V*_max_	Maximal reaction rate (U/mL)
*V*_B_	Molar volume of the solute (mL/mol)
*V*_S_	Molar volume of the solvent (mL/mol)
*x*	Microchannel length (mm)
*y*	Microchannel width (mm)
*Y*	FAME yield (%)
*W*	Half-width of microchannel (mm)

*Greek letters*

*ρ*	Solution density (kg/m^3^)
*η*_B_	Dynamic viscosity (kg/(m⋅s))
*ξ*	Dimensionless independent variables, *x*/*W*
*τ*	Residence time (s)
*τ*_D_	Diffusion time (s)
υ_ξ_	X-directional velocity (m/s)
*Φ*	Flow rate (µL/min)
*ψ*	Dimensionless independent variables, *y*/*W*

*Abbreviations*

aq	Aqueous phase
B	Buffer
E	Enzyme
FA	Fatty acids
i	Inlet
M	Methanol
MetOH	Methanol phase
S	Solute

## References

[1] Ma F., Hanna M.A. (1999). Biodiesel production: A review. Bioresour. Technol..

[2] Suurs A.A.R., Hekkert M.P. (2009). Competition between first and second generation technologies: Lessons from the formation of a biofuels innovation system in the Netherlands. Energy.

[3] Van Gerpen J. (2005). Biodiesel processing and production. Fuel Process. Technol..

[4] Gebremariam S.N., Marchetti J.M. (2017). Biodiesel production technologies: Review. AIMS Energy.

[5] Bart J.C.J., Palmeri N., Cavallaro S. (2010). Industrial process technology for biodiesel production. Biodiesel Science Technology Soil Oil.

[6] Issariyakul T., Dalai A.K. (2012). Comparative kinetics of transesterification for biodiesel production from palm oil and mustard oil. Can. J. Chem. Eng..

[7] Guldhe A., Singh B., Mutanda T., Permaul K., Bux F. (2015). Advances in synthesis of biodiesel via enzyme catalysis: Novel and sustainable approaches. Renew. Sust. Energ. Rev..

[8] Shah S., Sharma S., Gupta M.N. (2003). Enzymatic transesterification for biodiesel production. Indian J. Biochem. Biophys..

[9] Mittelbach M. (1990). Lipase catalyzed alcoholysis of sunflower oil. J. Am. Oil Chem. Soc..

[10] Christopher L.P., Kumar H., Zambare V.P. (2014). Enzymatic biodiesel: Challenges and opportunities. Appl. Energy.

[11] Teixeira C.B., Junior J.V.M., Macedo A. (2014). Biocatalysis combined with physical technologies for development of a green biodiesel process. Renew. Sust. Energ. Rev..

[12] Franjo M., Šalić A., Zelić B. (2018). Microstructured devices for biodiesel production by transesterification. Biomass Convers. Biorefin..

[13] Tonelli F., Evans S., Taticchi P. (2013). Industrial sustainability: Challenges, perspectives, actions. Int. J. Bus. Inov. Res..

[14] Budžaki S., Miljić G., Tišma M., Sundaram S., Hessel V. (2017). Is there a future for enzymatic biodiesel industrial production in microreactors?. Appl. Energy.

[15] Xie T., Zhang L., Xu N. (2012). Biodiesel synthesis in microreactors. Green Process. Synth..

[16] Mazubert A., Poux M., Aubin J. (2013). Intensified processes for FAME production from waste cooking oil: A technological review. Chem. Eng. J..

[17] Madhawan A., Arora A., Das J., Kuila A., Sharma V. (2018). Microreactor technology for biodiesel production: A review. Biomass Convers. Biorefin..

[18] Šalić A., Tušek A.J., Sander A., Zelić B. (2018). Lipase catalysed biodiesel synthesis with integrated glycerol separation in continuously operated microchips connected in series. New Biotechnol..

[19] Santana H.S., Silva J.L., Tortola D.S., Taranto O.P. (2018). Transesterification of sunflower oil in microchannels with circular obstructions. Chin. J. Chem. Eng..

[20] Mohammadi F., Rahimi M., Parvareh A., Feyzi M. (2017). Stimulation of magnetic nanoparticles to intensify transesterification of soybean oil in micromixers for biodiesel production. Chem. Eng. Process..

[21] Ulrich T., Borovinskaya E.S., Reschetilowski W. (2016). Versuchsplanerische Untersuchung der Umesterung von Sojaöl mit Ethanol im Mikroreaktor. Chem. Ing. Tech..

[22] Liu J., Chu Y., Cao X., Zhao Y., Xie H., Xue S. (2015). Rapid transesterification of micro-amount of lipids from microalgae via a micro-mixer reactor. Biotechnol. Biofuels.

[23] Zhou L., Lawal A. (2015). Evaluation of Presulfided NiMo/*γ*-Al_2_O_3_ for Hydrodeoxygenation of Microalgae Oil to Produce Green Diesel. Energy Fuels.

[24] Mukherjeea J., Guptab M.N. (2016). Dual bioimprinting of Thermomyces lanuginosus lipase for synthesis of biodiesel. Biotechnol. Rep..

[25] Shuit S.H., Ong Y.T., Lee K.T. (2012). Membrane technology as a promising alternative in biodiesel production: A review. Biotechnol. Adv..

[26] Qiu Z., Zhao L., Weatherley L. (2010). Process intensification technologies in continuous biodiesel production. Chem. Eng. Process..

[27] Kockmann N., Brand O., Fedder G.K., Hessel V., Renken A., Schouten J.C., Yoshida J. (2006). Micro Process Engineering—Fundamentals, Modeling, Fabrication, and Applications, Advanced Micro and Nanosystems. Handbook of Micro Reactors.

[28] Hessel V., Hardt S., Löwe H. (2004). Chemical Micro Process Engineering: Fundamentals, Modelling and Reactions.

[29] Budžaki S., Šalić A., Zelić B., Tišma M. (2015). Enzyme-catalysed Biodiesel Production from Edible and Waste Cooking Oils. Chem. Biochem. Eng. Q..

[30] Šalić A., Ivanković M., Ferk E., Zelić B. (2013). ADH based NAD^+^ regeneration in a microreactor. J. Chem. Technol. Biotechnol..

[31] Znidaršič-Plazl P., Plazl I. (2007). Steroid extraction in a microchannel system—mathematical modelling and experiments. Lab. Chip.

[32] Li J., Carr P.W. (1997). Accuracy of Empirical Correlations for Estimating Diffusion Coefficients in Aqueous Organic Mixtures. Anal. Chem..

[33] Young M.E., Carroad P.A., Bell R.L. (1980). Estimation of diffusion coefficients of proteins. Biotechnol. Bioeng..

[34] Yaws C.L. (1998). Chemical Properties Handbook: Physical, Thermodynamics, Environmental Transport, Safety & Health Related Properties for Organic & Inorganic Chemical.

[35] Zheng Y.Y., Guo X.H., Song N.N., Li D.C. (2011). Thermophilic lipase from *Thermomyces lanuginosus*: Gene cloning, expression and characterization. J. Mol. Catal. B Enzym..

[36] Akers H.A., Tuckler V.E. (1985). The Molar Volume of a Solute. Biochemistry and Molecular Biology Education.

[37] Poe S.L., Cummings M.A., Haaf M.P., McQuade D.T. (2006). Solving the clogging problem: Precipitate-forming reactions in flow. Angew. Chem. Ed..

[38] Liu S., Nie K., Zhang X., Wang M., Deng L., Ye X., Wang F., Tan T. (2014). Kinetic study on lipase-catalyzed biodiesel production from waste cooking oil. J. Mol. Catal. B Enzym..

[39] Tušek A., Šalić A., Kurtanjek Ž., Zelić B. (2012). Modelling and kinetic parameter estimation of alcohol dehydrogenase catalyzed hexanol oxidation in a microreactor. Eng. Life Sci..

[40] Firdaus M.Y., Guo Z., Fedosov S.N. (2016). Development of kinetic model for biodiesel production using liquid lipase as a biocatalyst, esterification step. Biochem. Eng. J..

[41] Cheirsilp B., H-Kittikun A., Limkatanyu S. (2008). Impact of transesterification mechanisms on the kinetic modeling of biodiesel production by immobilized lipase. Biochem. Eng. J..

[42] Sun S., Zhang L., Meng X., Xin Z. (2010). Kinetics of transesterification of palm oil and dimethyl carbonate for biodiesel production at the catalysis of heterogeneous base catalyst. Bioresour. Technol..

[43] Veny H., Aroua M.K., Sulaiman N.M.N. (2014). Kinetic study of lipase catalyzed transesterification of jatropha oil in circulated batch packed bed reactor. Chem. Eng. J..

[44] Patnaik P.R. (2011). On the dynamics of immobilized enzyme kinetics in a microreactor: A study of AP-catalyzed reactions. J. Biochem. Technol..

